# Young People’s Satisfaction With and Perceived Impact of a Multichannel Mental Health Helpline During and After COVID-19 Pandemic: Mixed Methods Analysis of Cross-Sectional Survey Data

**DOI:** 10.2196/68507

**Published:** 2026-02-03

**Authors:** Shyn Wei Phua, Anya Jan, Guanlin Zheng, Leslie Morrison Gutman

**Affiliations:** 1 Faculty of Brain Sciences, University College London, 1-19 Torrington Place, London, WC1E 7HB, United Kingdom, +44 7679-2000

**Keywords:** internet, online, adolescents, helpline, evaluation, qualitative, survey, quantitative, mental health, help-seeking

## Abstract

**Background:**

In the United Kingdom, there was an increased demand for young people’s mental health helpline services during COVID-19 pandemic, when face-to-face services were often inaccessible. Despite this, there is scant research examining young people’s experiences with these helplines during the pandemic and postpandemic periods.

**Objective:**

Using a cross-sectional survey, this mixed methods study aims to examine young people’s (aged 16-25+ years) experiences with the multichannel helpline provided by The Mix (Mental Health Innovations), the United Kingdom’s leading online mental health support service for young people during and after the pandemic.

**Methods:**

From February 2020 to October 2023, approximately 16,000 users aged 16-25+ years contacted The Mix’s helpline. All users were sent an email by The Mix following helpline contact to answer their user survey. Of these, 796 participants aged 16-25+ years answered the survey, representing a response rate of 5%, with a survey completion rate of 65.3%. To address potential nonresponse bias and missing data concerns, a multiple imputation procedure using the Multiple Imputation by Chained Equations (MICE) package in R (R Core Team) provided a final imputed sample for both the pandemic (n=295) and postpandemic (n=501) periods. Open-ended survey responses from users were also explored. Of the 796 participants who responded to the survey, there were 1183 open-ended responses from 486 respondents. Of these, a total of 731 open-ended responses (approximately 60% of the total responses) were coded. The criteria for inclusion were applied by 2 independent coders. Excluded responses focused on single words (eg, “thanks”), irrelevant text, or duplicated entries, ensuring only responses containing substantive feedback were analyzed.

**Results:**

During the pandemic, young people who contacted the helpline reported greater satisfaction after the first lockdown and a stronger perceived impact on their well‑being after the first lockdown and during the second and third lockdowns, compared with those who contacted the helpline during the gradual easing period; phone users reported higher satisfaction than those using the contact form. Postpandemic, helpline users who identified as “other” in terms of their gender reported less satisfaction, while male users reported a greater impact on their well-being compared to female users. Qualitative analysis revealed how the participants felt supported by the helpline, such as “feeling heard” and “being empowered,” and areas for improvement across service delivery, protocol, and technicalities.

**Conclusions:**

The findings highlight the important role that helplines play in supporting young people’s mental health, particularly in crises like the pandemic. This study underscores the need for service improvements to ensure young people continue to feel supported by helplines, highlighting key areas for improvement in service delivery, protocols, and technical infrastructure. Future research should explore channel preferences and minority experiences, often underrepresented in helpline studies.

## Introduction

### Background

Globally, COVID-19 pandemic led to significant mental health declines. In the United Kingdom, 24% of the population reported experiencing worsening distress between initial and subsequent waves of the pandemic [1]. Lockdown measures particularly affected young people, who, despite lower health risks, experienced declining mental health [2], especially compared to other age groups [3]. Even before the pandemic, existing literature highlighted a growing concern, an absence of critical precrisis support and significant difficulties accessing traditional mental health services for this age group [4-6]. Delays in accessing professional care have been linked to adverse psychological outcomes, underscoring the importance of timely intervention [7]. The pandemic exacerbated these issues, disrupting established mental health services and straining service capacity [8,9], highlighting the need for systemic reform and alternative support options [10].

In response to the increased need and lack of accessible support, there was a steep rise in demand for young people’s mental health helpline services during the pandemic [11]. A recent review [4] emphasized strong confidence in the evidence supporting the need for such crisis services staffed by trained professionals, especially those accessible via multiple platforms such as telephone, text, and email. Mental health helplines, which offer confidential, nonjudgmental, and often immediate support via telephone, web chat, and email, have been shown to be particularly valuable during a pandemic when face-to-face services and other supports are inaccessible [12]. Helplines are primarily staffed by trained volunteers equipped to provide emotional support without counseling and center around active listening and signposting to external support services [13]. The heavy reliance on helplines during crisis periods highlights their critical role as a timely accessible bridge to more comprehensive professional care.

Despite their increased use during the pandemic, there is scant research examining young people’s experiences with mental health helplines during the pandemic and postpandemic periods. Existing research often relies on call metrics (eg, volume and duration) rather than user-reported outcomes like satisfaction and perceived impact [14]. Methodologically, most studies are either purely quantitative or focus narrowly on crisis calls, with reviews noting short-term or proximal endpoints, heterogeneous measures, and limited attention to young people’s experiences [15]. This leaves a gap in understanding holistic user experience across nonphone channels (eg, chat and text). Such information can inform future service development and ensure helplines continue to meet the mental health needs of young people, especially during and after crisis periods.

To address these gaps, this current study examines cross-sectional survey data collected from 2020 to 2023 by The Mix (Mental Health Innovations), the United Kingdom’s leading online support service for young people under 25 years. The Mix operates a volunteer-based helpline focused on active listening and signposting, accessible through phone, email, web chat, and contact form. During the pandemic, The Mix saw a significant surge in demand, prompting them to expand their volunteer base and extend helpline hours to manage the increased contacts [11].

### Research Background

While randomized control trials are currently lacking, helplines seem to show benefits for young people seeking support for a range of issues, including depression and suicidality [16]. They may deescalate crises like self-harm and suicide [17], with one study in the United States demonstrating reduced psychological distress across demographic groups lasting up to 6 months after contact [17]. As community-based services appear more approachable than traditional professional services, helplines are well-positioned to mitigate the fear associated with seeking professional help, offering a valuable alternative [18]. Helplines’ timely, confidential, and user-directed nature further aligns with features that encourage help-seeking behavior in young people at risk of suicide [18]. One study [19] found that young adults report digital emotional support through texting or video calling as effective as in-person support for regulating negative emotions. As digital platforms become the norm for young people seeking support, helplines have the potential to overcome stigma and accessibility issues surrounding traditional services [20,21].

Globally, helplines experienced a sharp increase in usage during the pandemic. For instance, a study on Malta’s national mental health helpline reported a significant increase in calls, particularly during lockdown phases [22]. Samaritans UK reported an increased quantity and length of calls to the helpline, particularly between 2 AM and 6 AM, suggesting increased reliance on the service during lockdown periods [23]. A similar trend was observed in New Zealand, where young people were among the groups making more frequent contacts [24]. In Australia, Kids Helpline, which offers support via telephone, web chat, and email to children and young people, also saw an initial spike in demand, especially in the web chat modality, which remained the preferred mode of contact for young people throughout the pandemic [25]. The helpline recorded increases in contacts related to mental health, suicide, self-harm, and family relationship issues, with girls and adolescents aged 13‐18 years being the most frequent users. These findings regarding the growing concerns among youth helpline users echo a helpline report noting heightened contacts relating to anxiety, depression, and self-harm during the pandemic that have persisted in the postpandemic period [26]. This sustained and evolving reliance on helplines, coupled with the necessity of rapid operational changes to meet the demand, highlights the critical need to assess the user experience to ensure service quality and sustainability, an area currently lacking robust evidence.

Beyond the quantity of contacts, a significant shift in communication channels has emerged, with web chat and text-based services becoming increasingly popular among young people. An analysis of Kids Helpline Australia usage from 2012 to 2018 revealed that while phone and email contacts declined, web chat usage rose sharply [27]. Similarly, a mixed methods study of web-based suicide prevention services for young people in the United Kingdom found that young people prefer text-based communication over verbal channels when seeking support [21]. Despite this trend, several studies note that certain modalities, such as text-based services, are less examined, potentially due to insufficient funding [28,29]. Therefore, investigating young people’s experiences across nontelephone channels of delivery could provide valuable insight into their satisfaction with and perceived impact of the services. This line of inquiry is vital for optimizing resource allocation in the current digital service landscape.

Understanding the experiences of diverse helpline users is also crucial, as different groups present varying concerns and help-seeking behaviors. Research consistently shows that youth helpline users are predominantly female [26,27,30], a trend that mirrors patterns observed in general helplines [22]. During the pandemic, a German helpline reported a significant increase in calls, largely from female children and adolescents, who also experienced the most serious rise in serious mental health issues [31]. These trends underscore the importance of exploring whether satisfaction levels and perceived impact differ across genders among current helpline users, particularly between primary and underrepresented demographics.

Helpline services may require more nuanced tailoring to reach and support young people from diverse ethnic backgrounds. Although helplines are often considered beneficial for minority populations [14], a systematic review [28] identified a lack of evidence for youths in marginalized communities. This gap further underscores the need for more research exploring the helpline experiences of young people with diverse demographic characteristics, including age, gender, and ethnicity, to ensure equitable support.

### Conceptual Frameworks

To ground this study conceptually, we draw upon two interrelated bodies of literature: (1) help-seeking behavior models and (2) service evaluation frameworks. Help-seeking models conceptualize support-seeking as a multistage process shaped by perceived need, stigma, and accessibility [32]. Helplines, by offering anonymity and low-threshold access, play a pivotal role in overcoming structural barriers to formal care, particularly at the accessibility stage. Accordingly, this investigation into demographic differences and channel preferences is framed by the need to understand how service design influences successful engagement across diverse groups. Service evaluation frameworks further emphasize the importance of user-reported outcomes over operational metrics alone [33]. Together, these frameworks provide a lens through which to examine not only who engages with helpline services and how, but also whether those engagements translate into meaningful support experiences across diverse populations and modalities.

### This Study

Using cross-sectional survey data, this mixed methods study examines 2 datasets from The Mix’s mental health helpline services, one collected during the pandemic and the other in the postpandemic period. The study focuses on young people aged 16-25+ years and addresses 3 main research questions:

- What was young people’s satisfaction with the helpline services, and how did they perceive the services to impact their well-being during and after the pandemic?
- How did their satisfaction with and perceived impact of the helpline services vary by lockdown restrictions (pandemic dataset only), demographic characteristics (gender and ethnicity), and the channel through which support was accessed?
- Based on open-ended survey responses, how did young people describe feeling supported by the helpline, and what suggestions did they offer for improving the services?

## Methods

### Reporting Standards

This mixed methods study adheres to established reporting standards. Quantitative components were reported in accordance with the STROBE (Strengthening the Reporting of Observational Studies in Epidemiology) guidelines. Qualitative components were reported following the COREQ (Consolidated Criteria for Reporting Qualitative Research) checklist. Completed checklists are provided in Checklist 1 and Checklist 2.

### Participants

From February 2020 to October 2023, approximately 16,000 users aged 16-25+ years contacted the helpline. All users were sent an email by The Mix following contact to answer their user survey. Of these, 796 participants aged 16-25+ years answered the survey, representing a response rate of 5%, which is typical for these large-scale service evaluations [34]. Of the 796 participants, 520 answered questions about their satisfaction and perceived impact of the helpline, representing a completion rate of 65.3%, which is consistent with similar service evaluations [34]. Of the 520 participants, 257 answered the survey during the pandemic period (February 2020-April 2022) and 263 (May 2022-October 2023) answered the survey during the postpandemic period. Chi-square tests comparing the predictors and covariates indicate that those in the noncomplete sample were more likely to use email and less likely to use phone helplines compared to those in the complete sample (*χ*²_4_=41.846; *P*<.001). There were no other significant differences between the noncomplete and complete samples.

To address potential nonresponse bias and missing data concerns, we conducted a multiple imputation procedure using the Multiple Imputation by Chained Equations (MICE) package in R (R Core Team) [35]. Separate imputation models were specified for the during-pandemic and postpandemic datasets, including key sociodemographic and survey variables (eg, gender, ethnicity, channel, age, and outcome measures). Likert-type variables were imputed using proportional odds logistic regression (“polr”), categorical variables using multinomial logistic regression (“polyreg”), and continuous outcomes using predictive mean matching (“pmm”). Convergence diagnostics (using trace plots) and sensitivity analyses (eg, increasing the number of imputations and varying imputation methods) were performed to assess the robustness of the imputed results. The consistency of descriptive statistics across imputations indicated that non-response bias was unlikely to substantially affect the observed patterns.

Visual inspection of the trace plots across all 20 iterations confirmed that the multiple imputation models for the pandemic and postpandemic datasets achieved satisfactory convergence. Each colored trajectory represents an independent imputation chain. For most imputed variables, the mean and SD lines fluctuated modestly during the initial iterations before stabilizing around consistent central values, indicating that the Markov chains reached a stationary distribution. No systematic upward or downward trends were observed after iteration 10, and the chains demonstrated strong mixing with substantial overlap, suggesting good between-chain convergence and independence across imputations. Combined with sensitivity checks, these findings support the robustness and reliability of the imputed results for subsequent regression analyses [36].

Using the imputed sample, Table 1 provides the characteristics of the pandemic (n=295) and postpandemic (n=501) samples. Table 1 shows a clear demographic skew in the help-seeking population, participants self-identified as predominantly “female” and “White”. Age was concentrated in the 16‐18 years and 19‐21 years groups, with 22‐24 years declining postpandemic and 25+ years remaining a small proportion. This mirrors established trends in youth helpline literature [26,27,30] and indicates that the current findings are generalizable to The Mix’s majority user base. However, the underrepresentation of males and other groups (transgender, nonbinary, other, and prefer not to say), ethnic minorities (Asian, Black, Mixed, and Other), and older youth (25+ years) warrants caution in extrapolating results to these subgroups. In line with the help-seeking model, these distributional patterns may reflect differential barriers or preferences that shape who engages with standard helpline services.

**Table 1.** Participant characteristics of imputed pandemic and postpandemic samples.

Characteristics	Pandemic (N=295), n (%)		Postpandemic (N=501), n (%)	
Age (years)				
16‐18	105 (35.6)		190 (37.9)	
19‐21	72 (24.4)		170 (33.9)	
22‐24	97 (32.9)		100 (20)	
25+	21 (7.1)		41 (8.2)	
Sex				
Female	227 (76.9)		358 (71.5)	
Male	54 (18.3)		103 (20.6)	
Other	14 (4.7)		40 (8)	
Ethnicity				
White	227 (76.9)		387 (77.2)	
Minorities	68 (23.1)		114 (22.8)	
Helpline channel				
Contact form	51 (17.3)		5 (1)	
Email	154 (52.2)		192 (38.3)	
Phone	39 (13.2)		26 (5.2)	
Web chat	51 (17.3)		271 (54.1)	

### Procedure

From February 2020 to April 2022, survey invitations were sent a week following helpline contact. From May 2022 to October 2023, this was shortened to one day, with a reminder sent the following week if the survey was not completed. Participants completed the online survey on SmartSurvey (SmartSurvey is Smartline International Ltd).

### Measures

#### Outcomes

The response scale changed from a 5-point (1=Strongly disagree; 2=Disagree; 3=Neutral; 4=Agree; 5=Strongly agree) to a 4-point scale (1=Strongly disagree; 2=Tend to disagree; 3=Tend to agree; 4=Strongly agree) from the pandemic to postpandemic periods. In both versions, “Not relevant” and “Don’t know” options were also included. For this reason, the pandemic and postpandemic periods were examined separately in 2 different models; thus, each of their reliabilities is reported. Factor analysis confirmed a 2-factor model was an adequate fit to the data (Pandemic: comparative fit index [CFI]=0.97; Tucker-Lewis Index [TLI]=0.95; root mean square error of approximation [RMSEA]=0.13; standardized root mean square residual [SRMR]=0.04 and Postpandemic: CFI=0.98; TLI=0.96; RMSEA=0.10; SRMR=.04). Satisfaction was defined as participants’ perceptions of service quality, while Impact was defined as the effectiveness of the service in positively influencing well-being or behavior.

#### Satisfaction

Cronbach alphas showed high reliability (α=.94 pandemic; .90 postpandemic). The mean of 3 questions was computed, assessing participants’ satisfaction with the service. Questions included “I felt listened to,” “I felt understood,” and “I felt emotionally supported.”

#### Perceived Impact on Well-being

Cronbach alphas showed high reliability (α=.93 pandemic; .93 postpandemic). The mean of 4 questions was computed, which assessed perceived changes in participants’ well-being and behavior. Questions included “My wellbeing improved,” “I feel more able to cope with my situation or issue,” “I feel more capable to make decisions,” and “I formed/I will form a plan to make a positive change to my situation.”

### Predictors and Covariates

#### Time Period

**Figure 1.**
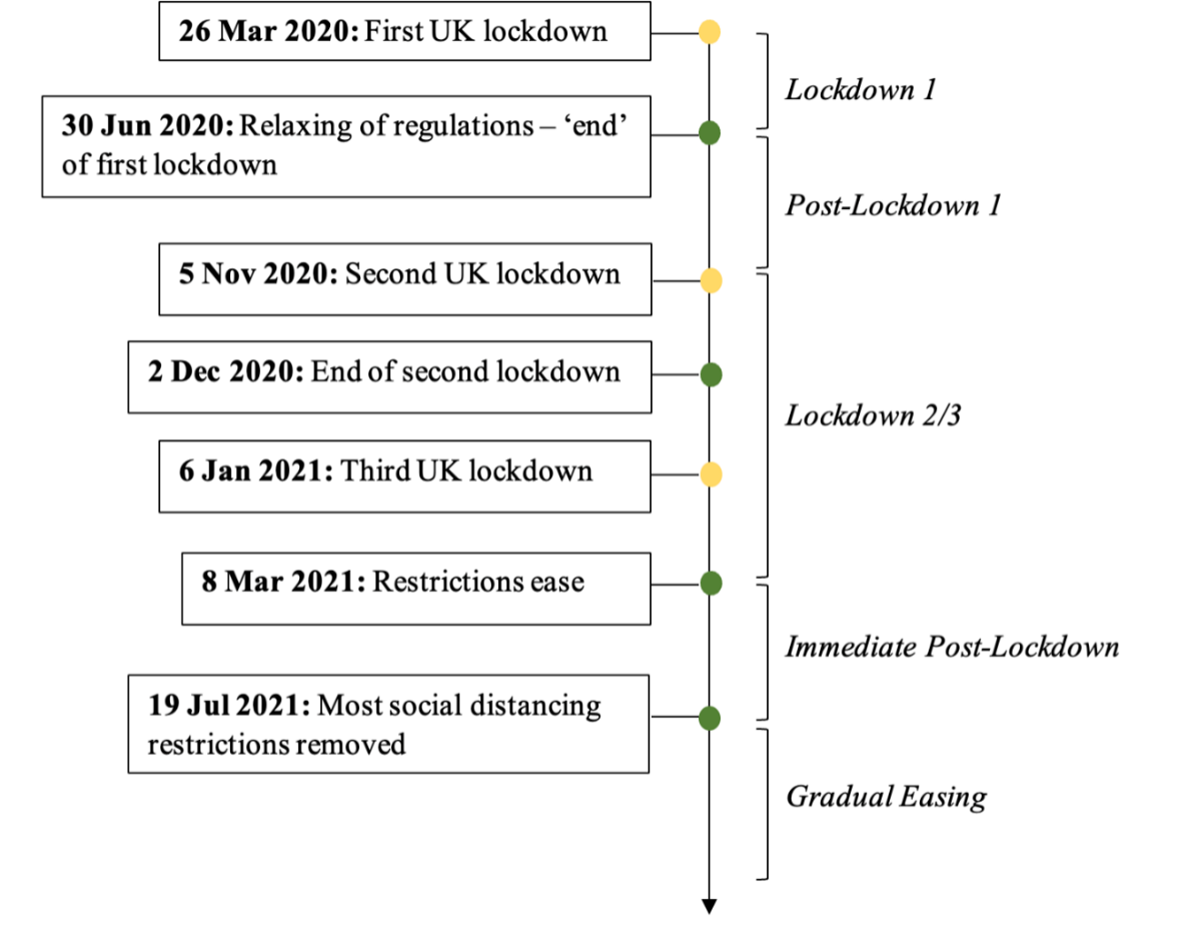
Timeline of COVID-19 pandemic restrictions from March 2020 to July 2021 in the United Kingdom.

The time and date of the helpline contact was recorded by The Mix. Using this information, 5 time periods were created to reflect the United Kingdom’s COVID-19 pandemic restrictions from the Institute for Government (2022), including Lockdown 1 (March 2020-June 2020), Postlockdown 1 (June 2020-November 2020), Lockdown 2/3 (November 2020-March 2021), Immediate postlockdown (March 2021-July 2021), and gradual restriction easing (July 2021-April 2022, reference category) (see Figure 1). For the postpandemic model, the date was included as a continuous control variable (months since Lockdown 1). Consistent with this framework, we designated satisfaction and perceived impact as primary outcomes in a remote, multichannel environment where voice cues and interpersonal warmth may be diminished [33]. We additionally analyzed young people’s open-ended responses to provide user-centered explanations of how service variables, such as channel delivery and time period, related to perceived outcomes.

#### Age

Service users were asked their age during helpline contact by The Mix. Their responses were coded into 5 categories 16‐18 years (reference category), 19‐21 years, 22‐24 years, 25+ years, and didn’t ask/say.

#### Gender

Service users were asked their gender during helpline contact by The Mix. Their responses were coded into 4 categories: female (reference category), male, other, and didn’t ask/say.

#### Ethnicity

Service users were asked their ethnicity during helpline contact by The Mix. Due to small numbers for the ethnic minorities (see Table 1), their responses were combined into a single group for analysis. There were 3 categories: White (reference category), ethnic minority, and didn’t ask/say.

#### Helpline Channel

The helpline channel was recorded by The Mix. There were 4 helpline channels, including phone, web chat, email, and contact form (reference category).

#### Overall Helpline Rating

Participants responded to the question, “How would you rate the helpline service overall?” (1=Very poor; 5=Excellent).

### Open-Ended Questions

Qualitative data were obtained from three open-ended questions, “Please tell us how the service helped you,” “What could we have done to support you even better?*”* and “How can we improve?*”*

### Data Analysis

#### Quantitative Analysis

Statistical analysis was performed using R software (version 4.3.1; R Core Team). Multiple linear regression models were used to examine satisfaction and perceived impact across the fixed predictor of time, controlling for covariates. Due to the different response scales for the outcomes, models were run separately for the pandemic and postpandemic periods. Specifically, the Likert scale for satisfaction and perceived impact was modified from a 5−point scale (used in the pandemic period) to a 4−point scale (used in the postpandemic period) to simplify completion. Four regression models were constructed (2 outcomes x 2 datasets). The same reference group was selected for each categorical predictor across both datasets, including female (sex), White (ethnicity), and 16-18 years (age). For the Helpline Channel, the largest category differed between datasets, so Contact Form, the second largest in both, was chosen as the reference. For the pandemic dataset, the gradual easing phase was the reference. Interactions between time period and predictors and between demographic variables and channel use were explored in the regression models but were either nonsignificant or could not be examined due to insufficient sample sizes in each category and were therefore excluded from the final models.

#### Qualitative Analysis

Open-ended responses varied significantly in length and quality. To ensure meaningful thematic analysis, responses were first excluded if they met any of the following criteria (1) illogical, (2) overly vague, (3) “N/A” or “I don’t know” response without elaboration. Additionally, responses solely discussing signposting were excluded as these were analyzed by another researcher on the project; our focus was the overall helpline service. Responses that indicated “no improvements necessary” (n=161) or “service did not help” (n=8) without further elaboration were also excluded, as our analysis centered on how participants felt helped and actionable insights for improvement. Of the 1183 qualitative responses, 452 responses were removed according to the exclusion criteria (40% of the total responses). In total, there were 731 responses from 486 out of 796 respondents, with approximately 60% of the sample providing at least one open-ended response.

Following the thematic analysis guidelines [29], an initial review of responses involved “active reading,” where potential codes were noted. Inductive coding was then conducted in NVivo 14 (QSR International), focusing on what users found helpful or unhelpful about the service. Codes were organized into overarching themes and subthemes to ensure significance and distinctiveness. A codebook (in Section S2, Multimedia Appendix 1) was created and used for a final round of deductive coding in Microsoft Excel [32], with each response assigned up to four codes. There were disproportionately more responses from the postpandemic period (n=562) compared to the pandemic period (n=169).

Using the codebook, a second coder independently coded 20% of responses at random (n=152). Percent agreement, representing the proportion of cases where at least one code assigned matched between coders, was calculated at 87.6%. This prominent level of interrater reliability indicated consistent coding practice, enhancing the reliability of the study’s findings.

### Ethical Considerations

This study was approved by the University College London (UCL) Research Ethics Committee (reference 16583/003 on July 27, 2023). Participants were provided with informed consent before completing the online survey. Survey data were fully anonymized. As an incentive for participation, participants were entered into a lucky draw with the chance to win a £50 ($66.64) Amazon voucher.

## Results

### Quantitative Analysis

After imputation, in the pandemic sample, the mean of user satisfaction was 3.73 (SD 1.22) and the mean of perceived impact was 3.21 (SD 1.08), indicating responses between “neutral” and “agree”; in the postpandemic sample, the mean of user satisfaction was 3.31 (SD 0.87), indicating a response between “tend to agree” and “strongly agree”, and the mean of perceived impact was 2.76 (SD 0.87), indicating a response close to “tend to agree”.

#### Satisfaction and Perceived Impact During the Pandemic

Table 2 shows the statistical coefficients and significance for satisfaction with the helpline service during the pandemic period. Satisfaction was significantly greater during postlockdown 1 than during the gradual easing phase. Phone users reported higher satisfaction than contact form users, and higher overall helpline ratings were linked to greater satisfaction. The model explained 54.7% of the variance.

**Table 2.** Regression model for user satisfaction of The Mix’s mental health helplines during the COVID-19 pandemic.

Predictors	Estimate	SE	95% CI	*P* value
Intercept	0.44	0.27	–0.09 to 0.97	.10
Period
Lockdown1	0.29	0.16	–0.03 to 0.62	.07
Postlockdown1	0.32	0.16	–0.00 to 0.64	.05
Lockdown 2/3	0.16	0.23	–0.31 to 0.64	.49
Immediate postlockdown	0.26	0.16	–0.06 to 0.58	.10
Sex
Male	–0.11	0.14	–0.38 to 0.15	.40
Other	0.11	0.28	–0.45 to 0.68	.68
Ethnicity (minorities)	–0.07	0.12	–0.31 to 0.18	.58
Age (years)
19-21	0.09	0.13	–0.16 to 0.34	.48
22-24	0.11	0.14	–0.17 to 0.40	.43
25+	0.28	0.19	–0.10 to 0.66	.15
Channel
Email	0.06	0.14	–0.22 to 0.34	.69
Phone	0.44	0.19	0.06 to 0.83	.02
Web chat	0.22	0.20	–0.20 to 0.64	.29
Overall helpline rating	0.76	0.05	0.66 to 0.86	<.001
*R*^2^ adjusted	0.547	—^a^	—	—

a Not applicable.

Table 3 shows the statistical coefficients and significance for perceived impact on well-being for the helpline service during the pandemic period. Perceived impact was significantly greater during postlockdown 1 and lockdowns 2/3 than during the gradual easing phase. Higher overall helpline ratings were also linked to greater perceived impact. The model explained 50.8% of variance.

**Table 3.** Regression model for perceived impact on well-being of The Mix’s mental health helplines during the COVID-19 pandemic.

Predictors	Estimate	SE	95% CI	*P* value
Intercept	0.39	0.24	–0.08 to 0.86	.10
Period
Lockdown1	0.24	0.16	–0.08 to 0.55	.14
Postlockdown1	0.41	0.16	0.10 to 0.73	.01
Lockdown 2/3	0.46	0.18	0.10 to 0.81	.01
Immediate postlockdown	0.14	0.15	–0.16 to 0.43	.36
Gender
Men	0.16	0.14	–0.11 to 0.43	.24
Other	0.31	0.27	–0.24 to 0.86	.25
Ethnicity (minorities)	–0.16	0.13	–0.43 to 0.11	.23
Age (years)
19-21	0.02	0.13	–0.23 to 0.27	.87
22-24	0.19	0.12	–0.04 to 0.42	.11
25+	0.31	0.23	–0.17 to 0.79	.20
Channel
Email	–0.02	0.15	–0.31 to 0.27	.89
Phone	0.01	0.21	–0.41 to 0.43	.95
Web chat	–0.28	0.19	–0.66 to 0.10	.14
Overall helpline rating	0.66	0.04	0.57‐0.74	<.001
*R*^2^ adjusted	0.508	—^a^	—	—

a Not applicable.

#### Satisfaction and Perceived Impact Postpandemic

Table 4 shows the statistical coefficients and significance for satisfaction with the helpline service postpandemic. Participants who identified as “other” in terms of their gender reported lower satisfaction than females. Overall helpline rating predicted greater satisfaction. The model explained 39.6% of variance.

**Table 4.** Regression model for user satisfaction of The Mix’s mental health helplines postpandemic.

Predictors	Estimate	SE	95% CI	*P* value
Intercept	2.01	0.48	1.03‐2.99	<.001
Months since lockdown 1	–0.005	0.007	–0.02 to 0.01	.53
Sex
Male	0.02	0.11	–0.21 to 0.24	.88
Other	–0.40	0.13	–0.67 to –0.13	.005
Ethnicity (minorities)	0.00	0.09	–0.19 to 0.19	.99
Age (years)
19–21	–0.03	0.09	–0.21 to 0.15	.74
22–24	–0.11	0.11	–0.33 to 0.11	.32
25+	–0.13	0.18	–0.52 to 0.26	.48
Channel
Contact form	–0.51	0.38	–1.28 to 0.26	.19
Email	–0.62	0.37	–1.36 to 0.12	.10
Phone	–0.39	0.47	–1.34 to 0.55	.41
Web chat	–0.38	0.38	–1.15 to 0.39	.32
Overall helpline rating	0.49	0.04	0.42‐0.56	<.001
*R*^2^ adjusted	0.398	—^a^	—	—

a Not applicable.

Table 5 shows the statistical coefficients and significance for perceived impact on well-being for the helpline service postpandemic. Participants who identified as male reported greater impact than females. Overall helpline rating predicted greater perceived impact. The model explained 41.6% of variance.

**Table 5.** Regression model for perceived impact on well-being of The Mix’s mental health helplines post-pandemic.

Predictors	Estimate	SE	95% CI	*P* value
Intercept	0.62	0.40	–0.16 to 1.41	.12
Months since lockdown 1	0.003	0.01	–0.01 to 0.02	.65
Sex
Male	0.23	0.08	0.07‐0.40	.005
Other	–0.08	0.16	–0.42 to 0.25	.60
Ethnicity (minorities)	0.11	0.09	–0.08 to 0.30	.24
Age (years)
19–21	0.04	0.08	–0.12 to 0.19	.65
22–24	–0.11	0.13	–0.41 to 0.18	.41
25+	0.13	0.12	–0.10 to 0.36	.27
Channel
Contact form	–0.14	0.35	–0.85 to 0.56	.69
Email	–0.19	0.35	–0.89 to 0.50	.58
Phone	–0.08	0.43	–0.94 to 0.78	.85
Web chat	–0.01	0.35	–0.70 to 0.68	.98
Overall helpline rating	0.50	0.03	0.44‐0.57	<.001
*R*^2^ adjusted	0.416	—^a^	—	—

a Not applicable.

### Qualitative Analysis

#### Open-Ended Responses From Participants

There were 4 overarching themes, one theme focused on types of support and 3 themes focused on improvements involving service delivery, service protocol, and service technicalities. No differences in themes were noted between the pandemic and postpandemic periods. Table 6 provides a definition of each theme and subtheme, along with the number of times it was mentioned in the open-ended responses.

**Table 6.** Thematic analysis of users’ open-ended survey responses about The Mix’s mental health helpline (N=731).

Theme	Theme definition	Subtheme	Subtheme definition	Frequency of mentions
Types of support (n=417)	Ways in which users felt helped (if they did) by the service.	Felt heard Someone to talk to Better understanding of emotions and circumstances Empowerment and direction Timely support	Felt listened to and supported without judgment Felt less lonely or benefitted from talking through their issues with someone Made sense of their emotions, or gained new perspective regarding their situation Felt more confident, optimistic, and clear on how to seek help or move forward from their situation Acute and timely support, practically (eg, emergency services), or emotionally (eg, calming down)	174 85 30 62 66
Service delivery (n=308)	Sentiments regarding the effectiveness of helpline delivery and execution by volunteers.	Delivery and tone Conversational elements Rushing and rejection Responsiveness	Pace, tone, and phrases used by volunteers during conversation with users Effectiveness of questions asked by volunteers and conversational practices (eg, rephrasing and repeating) Sentiments of feeling pushed away, unheard, misunderstood, or excluded How quickly, or whether, users received responses from volunteers (wait times)	73 38 99 98
Service protocol (n=105)	Sentiments regarding service protocol and standard practice by which the helpline is run.	Lack of personal input and concrete support Alternative support Talk times	Lack of personal opinions from volunteers and practical advice regarding solutions or action plans Preference for longer-term follow-up support or face-to-face support Preference for longer talk times and less restriction on chat duration	66 23 16
Service technicalities (n=80)	Sentiments regarding technical and administrative aspects of the service, including opening hours, IT matters, and advertisement.	Opening hours Platform functionality Advertising	Preference for keeping the helpline open for extended periods of time Ensuring platforms are running without technical issues, and suggestions for improving platform functionality Extending advertising of the service to wider audiences, and providing better/clearer information on how the service works	18 47 15

#### Types of Support

The most prevalent themes of support highlighted participants feeling heard and having someone to talk to.

##### Felt Heard

Participants reported feeling heard, supported, and cared for (*“*helped…knowing these people obviously care…[they] hear for us and support if I reach out…[this] comforted me*”*). Some highlighted the importance of patience and nonjudgmental attitudes of volunteers (*“*never felt judged or rushed*”*) and empathetic efforts (*“*counselors were trying their best to understand my situation*”*). This made them feel respected and validated (*“*felt…respected…[reassured] me that my feelings were valid*”*), creating a safe space for seeking comfort (*“*a space where I felt listened to and valued*”*).

##### Someone to Talk To

Many participants underscored the value of having someone to talk to, which made them feel less alone (*“*felt someone was with me when I’m alone”). They also noted the relief (“[got] things off my chest”) and perspective (“helped me to really take a step back and see more clearly”) gained from talking through their situation with a third party.

##### Better Understanding of Emotions and Circumstances

Some participants mentioned that talking to volunteers helped them “make sense of [their] feelings,” “see the situation more clearly,” and “understand [their] problems better.”

##### Empowerment and Direction

Participants occasionally mentioned feeling more optimistic (*“*I now have a positive outlook on life*”*) and hopeful (*“*a sense of hope and some pleasure towards the future*”*) after contacting the helpline. Some also expressed feeling clearer about their options (*“*[understood] what I could do next*”*) and empowered to act (*“*Helped me reach out to getting help”; “I felt less distressed and had a plan how to go forward*”*).

##### Timely Support

Several participants emphasized the timely intervention received, both practically (*“*They knew I was in danger of myself and got me immediate help from the police”) and emotionally (*“*[calmed] me down when I was feeling very distressed”; “Distracted me from my thoughts”). Notably, however, a few participants expressed intense dissatisfaction with contacting emergency services (*“*[Don’t] call the emergency services and panic my family*”*).

### Service Delivery

The most common themes focused on responsiveness, along with participants’ experiences of feeling rushed or rejected by the service.

#### Delivery and Tone

A major concern was the robotic tone of volunteers. Some participants reported scripted and impersonal conversations (“[feels] like I’m speaking to a chat bot…always the same phrase”; “language seemed like…a generic template…difficult to feel understood”). Several participants suggested being “less formal,” being “a little more personal / [comforting],” and adding “some encouragement.” Participants who felt volunteers’ compassion felt heard and supported (“I felt emotionally supported…extremely thoughtful and compassionate”).

#### Conversational Elements

Some participants found conversations monotonous as volunteers frequently repeated what they said (*“*Perhaps not echo what I say back…[gets] a bit repetitive”). Additionally, some participants expressed preferences to be asked more questions, particularly regarding “roots/sources of problems, feelings or thoughts.”

#### Rushing and Rejection

Participants who felt rushed by volunteers felt unheard (*“*[could have] listened to what I was saying instead of trying to quickly end the chat*”*). One participant highlighted the need for improved volunteer training, as “it was all rushed and the workers “needed” to go…didn’t take me seriously.” A similar issue with signposting was raised, with some feeling unsupported (“didn’t really receive…support, just the links…without giving much more guidance”) and pushed away (“Not asked me to go somewhere else”).

#### Responsiveness

Participants frequently expressed frustration with slow response times (“reply time could be a bit quicker”) or receiving no response (“no one got back to me”). Email users seemed particularly dissatisfied, noting neglect of follow-up emails (“actually respond to my second email???”). However, a few participants reported prompt responses (“far quicker than I thought”), which were crucial for vulnerable individuals (*“*responses…was hasty, which was important in my state at the time”).

### Service Protocol

The most prominent theme centered on volunteers’ limitations in offering personal input and practical support.

#### Lack of Personal Input and Concrete Support

A portion of participants expressed preferences for receiving input from volunteers (“Would’ve appreciated more input from the agent…repetition of my messages…made me feel worse”). Additionally, several participants suggested providing practical support in the form of grounding techniques and action plans (“[Teach] breathing exercises”; “More help with creating a plan of action”).

#### Alternative Support

Some participants commented that follow-up checks (“Check in a few days after to see if things improved”) and offline support (“an office people can go to”) could be valuable improvements to current support.

#### Talk Times

A small subset of sentiments emerged postpandemic, expressing preferences for longer conversations (“Let people talk for longer”) and frustrations with talk-time restrictions (“didn’t even finish the conversation …time ran out which frustrated me”).

### Service Technicalities

The most prevalent theme related to platform functionality issues, along with suggestions for improvement.

#### Opening Hours

Several participants suggested extended opening hours would be useful (“have the web chat open longer on the weekends and during the week”), especially into the night (“Keep the lines open till 12”).

#### Platform Functionality

Some participants encountered technical web chat issues (“[it] cut off”). Others highlighted that “the chat [was] offline even during open hours.” Suggestions included providing pre-emptive notifications if the helpline was busy (“helpful…to have made it clear on the site that this might sometimes happen”). Other proposed improvements included implementing a *“*traffic light system” for waiting and providing “more specific” information about queue status.

#### Advertising

A few participants highlighted the need for clearer advertising regarding the services offered, suggesting to “mention on the website what to expect” and “the time period [given]*.*” Additionally, there were suggestions for increased promotion to reach a wider audience (“more promotion…dug quite deep to find this...more people could use this”).

## Discussion

### Principal Findings

Using cross-sectional survey data collected by The Mix from 2020 to 2023, this mixed methods study provides insights into how young people’s experiences with the services varied according to lockdown restrictions (pandemic period only), their demographic characteristics, and the channel of delivery used to access the services. During the pandemic, young people’s average responses ranged from “neutral” to “agree” regarding their satisfaction with the helpline service and their perception that it supported their well-being. In this period, young people reported greater satisfaction with the helpline postlockdown 1, and a stronger impact on their well‑being postlockdown 1 and during lockdowns 2 and 3 compared with the period of gradually easing restrictions; phone users also reported higher satisfaction with the helpline compared to contact form users. Postpandemic, young people’s average satisfaction with the helpline service exceeded the “tend to agree” threshold, while their perception that it supported their well-being was slightly below that level. In this period, helpline users who identified as “other” in terms of their gender reported lower satisfaction, while male users reported greater impact compared to female users. Thematic analyses offered deeper insight into ways in which participants felt supported and identified areas where participants felt there could be improvement.

### Satisfaction and Perceived Impact During the Pandemic and Postpandemic

These findings—higher satisfaction after the first lockdown and greater perceived impact after the first, second, and third lockdowns compared with the gradual easing period—may reflect the additional measures The Mix implemented to meet increased demand. These measures included recruiting more volunteer counselors and extending the helpline’s operational hours by 2 hours each day. As a result, The Mix’s helpline saw a 14% increase in the number of contacts accepted since March 2020 [11], in line with evidence of pandemic-related surges in helpline demand across services [37]. This highlights the importance of helpline services being responsive to the greater mental health needs of young people during crisis periods.

This study also revealed differences in users’ reported outcomes according to helpline channel. During the pandemic, phone users reported higher satisfaction than contact form users, potentially due to the nature of interaction. One helpline study [38] underscored the value of human connection in helpline support, where a volunteer’s voice can convey warmth and reassurance, an element absent in contact forms. This verbal support could have been especially valuable during a time of heightened loneliness and social isolation for young people [13], especially as there was no difference in reported outcomes between channels during the postpandemic period.

In terms of demographic characteristics of the users, the sample remained predominantly female and White across periods. Postpandemic, there was a modest shift in gender composition, with the proportion of males increasing to 20.6% and those who identified as “other” in terms of their gender increasing by 8%. In postpandemic models, participants identifying as “other” reported lower satisfaction than females, while males reported greater perceived impact than females. The reasons explaining these statistical trends are unclear, however. Nevertheless, absolute numbers for some groups remain comparatively small—particularly minority ethnic youth (22.8% postpandemic) and the “other” gender category—limiting precision for subgroup and intersectional analyses; larger, targeted samples are needed to draw firmer conclusions, along with in-depth qualitative interviews to understand quantitative patterns.

While helplines have been recognized for their role in supporting minority groups, including Black and minority ethnic individuals, who are less likely to seek support through traditional avenues [15,39], our findings did not reveal any ethnic differences in satisfaction or perceived impact during the pandemic or postpandemic periods. This may be due to the small numbers of minority-ethnic respondents, which limited statistical power and widened confidence intervals, making modest differences hard to detect.

As expected, a strong relationship emerged between the young people’s overall helpline rating and their reported satisfaction and perceived impact of the helpline during both the pandemic and postpandemic periods. When users had a positive overall experience, they reported higher satisfaction levels and felt the service had a greater impact on their mental well-being. This finding underscores the importance of understanding young people’s experiences with the helpline, as these perceptions are key to supporting their mental health, in line with evidence that perceived helpfulness/satisfaction is associated with immediate reductions in distress and with the perceived effectiveness of crisis interventions [40]. Qualitative data offers further insight into these experiences. The next section explores key themes from young people’s open-ended responses, linking these insights to the quantitative findings. It highlights their preferences for support and identifies opportunities to improve services.

### Open-Ended Responses From Participants

Our thematic analysis highlighted multi-faceted benefits of the helpline for young people facing mental health challenges, aligning with previous findings [15]. Participants valued feeling heard and having someone to talk to and reported improved coping abilities and clarity about their emotions and circumstances. These benefits were described to alleviate loneliness, distract from unhealthy tendencies, and prompt further help-seeking behavior in the future with users of an eating-disorder helpline. Such support may have been particularly valuable in the context of social isolation during the pandemic, reflected in the quantitative findings showing higher perceived impact and a preference for the telephone helpline versus the contact form during this period. Additionally, while opinions on the involvement of emergency services were mixed, participants appreciated timely intervention by the helpline, ensuring participants were supported during emotional distress and kept safe from dangerous behaviors.

Nonetheless, participants identified several areas for improvement within the helpline service. Many emphasized the need to address variable service delivery standards, noting that some interactions felt robotic and impersonal, and they sometimes felt rushed and rejected by volunteers. This feedback aligns broader user perspectives on helpline services, which highlight the importance of a volunteer’s engagement—especially through active listening—as crucial for user satisfaction and positive outcomes [41]. Participants’ strong preference for a service that feels more personal and empathetic suggests that the higher satisfaction reported by phone users may stem from voice-based interactions, which likely conveyed more warmth and reassurance through vocal cues [42].

Concerns regarding service availability and responsiveness were also significant factors for young people seeking immediate support. Participants mentioned preferences for extended operating hours, aligning with research indicating user preferences for round-the-clock availability [43]. One suggested focusing on critical times, which could be after 11 PM when young people are more likely to seek help online [44,45]. Additionally, implementing a traffic light system on the website to indicate wait times was recommended, which could alleviate anxiety and frustration during busy periods by providing clearer estimates [46].

Young people also expressed the need for wider and clearer advertising to reach potential users. The volunteer-staffed nature of The Mix is central to its ability to provide low-threshold, nonclinical support. However, this model warrants critical evaluation in the context of outcomes. Future service evaluations should include operational metrics on volunteer retention and training consistency and link these directly to user-reported outcomes to ensure the model remains both scalable and high-quality.

Although the helpline was recognized as a valuable resource, participants noted it was not easily accessed or well-promoted. Transparency regarding the helpline’s role was also emphasized, particularly the need to communicate that personal advice cannot be provided, as many were disappointed by the lack of advice provided during their experience. The idea of offering practical support was mentioned. For instance, a few participants suggested providing grounding techniques, which can be delivered by nontherapists [47]. Although mentioned less frequently, the exploration of follow-up and in-person support options could be valuable in addressing unmet needs for those seeking more comprehensive assistance.

### Limitations

This study has several limitations, which must be considered in light of the findings. First, the sample was predominantly female and White, potentially underrepresenting males and minorities. Due to the small sample size, specific gender and ethnic groups were collated and not examined separately. Additionally, chi-square tests indicated differences in helpline channel usage between excluded and included participants, suggesting potential underrepresentation of email users, especially in the postpandemic sample. Survey representativeness is a common issue in online helpline research due to confidentiality, limiting sampling strategies [27]. Nevertheless, this study highlights the tendency to overlook underrepresented groups in helpline research, aligning with existing critiques [28], emphasizing the significant need for further research focused on these populations. Addressing this gap is crucial, as these groups are frequently described as having both the potential and the need to benefit from such services [15].

Another key limitation of this cross-sectional study is its reliance on a self-administered survey embedded within a service context, which yielded a low completion rate across the full user population. This introduces a substantial risk of non-response bias, potentially limiting the generalizability of our findings. Although we addressed missing data using MICE, we acknowledge that the absence of statistical weighting constrains our ability to fully account for differential response patterns.

Furthermore, the regression models were limited by the reliance on service data, which had several limitations, including the lack of psychometrically robust measures and volunteer bias. This also meant a lack of key clinical confounders (eg, prior mental health history, clinical severity, or specific contact reason) to include in the statistical models. Consequently, the findings should be viewed as exploratory associations. Finally, a major methodological constraint is the nonstandardized change in the Likert scale for satisfaction and impact (from a 5-point to a 4-point scale) between the two time periods; this difference precludes direct statistical comparison of mean scores across the pandemic and postpandemic periods.

In the qualitative component, the systematic exclusion of a large proportion of open-ended responses, while necessary to maintain the integrity of the thematic analysis, is a source of potential selection bias, which may restrict the diversity and trustworthiness of the qualitative themes. Similarly, qualitative data was unevenly distributed between pandemic and postpandemic periods, potentially underrepresenting pandemic-period sentiments. This uneven distribution made it difficult to map thematic insights to specific periods, limiting our understanding of the most relevant issues. Future research should involve in-depth interviews with purposefully sampled users to better understand their expectations and experiences with the helpline services and their suggestions for improvement.

Finally, the lack of prepandemic data limited our ability to compare helpline experiences across prepandemic, pandemic, and postpandemic periods. Such comparisons could have illuminated the pandemic’s impact on helpline experiences and whether user satisfaction and impact returned to prepandemic levels or established a new normal. Finally, the reliance on self-report for both satisfaction and perceived impact means the observed correlation may be inflated due to shared method variance, warranting caution in their interpretation.

### Conclusions

This study highlights the perceived positive impact of helplines in supporting young people’s mental health, particularly during the pandemic, which saw surges in contacts surrounding anxiety, depression, and loneliness [26] alongside disrupted mental health services. During the pandemic, phone calls, offering a more personal connection, were linked to greater satisfaction than contact forms. These findings suggest a need for further research into channel preferences to align services with users’ needs better and optimize resource allocation. Qualitative themes contextualized the quantitative patterns: young people highlighted timeliness, continuity, channel fit, and empathic, validating conversations as central to feeling helped, suggesting concrete targets for service improvement.

Key areas for improvement were identified, including service delivery, protocol, and technicalities. Based on the qualitative feedback regarding access difficulties during crises and the observed decline in postpandemic satisfaction, mental health helplines for young people should prioritize extending hours of operation for digital channels to ensure service continuity during nontraditional hours, alongside implementing a clearer queue system as suggested by young people. The frequent qualitative theme regarding the need for warmer and less scripted communication directly informs the need to enhance volunteer training in reflective listening and emotional validation, particularly for web chat modalities where nonverbal cues are absent. Addressing these areas is vital to ensuring helplines remain an accessible bridge to more comprehensive mental health support, thus improving overall experiences and enhancing satisfaction and perceived impact. Overall, the findings reinforce the value of helplines as interim support for young people awaiting professional care, offering critical, emotional, and informational support in times of need.

## Supplementary material

10.2196/68507 Multimedia Appendix 1 Helpline feedback survey from The Mix.

10.2196/68507 Checklist 1 Strengthening the Reporting of Observational Studies in Epidemiology checklist v4 combined Plos Medicine.

10.2196/68507 Checklist 2 Consolidated Criteria for Reporting Qualitative Research checklist.

## Abbreviations

COREQ: Consolidated Criteria for Reporting Qualitative Research
MICE: Multiple Imputation by Chained Equations
STROBE: Strengthening the Reporting of Observational Studies in Epidemiology
