# Supplementary material for: Young People’s Satisfaction With and Perceived Impact of a Multichannel Mental Health Helpline During and After COVID-19 Pandemic: Mixed Methods Analysis of Cross-Sectional Survey Data
Source: JMIR Form Res. 2026 Feb 3;10:e68507. doi: 10.2196/68507 (PMC12872607; doi:10.2196/68507)
Supplement: Multimedia Appendix 1 [file formative-v10-e68507-s001.docx]

# Section S1

Helpline Feedback Survey from The Mix

**1. Introduction**

**Thank you for contacting The Mix helpline in the last few days. We are always thinking about improving our services. Please complete this short survey about your experience with The Mix helpline to help us understand how we can improve.

Take this short survey and you could win a £50 Amazon voucher​.

Everything you say is confidential and held in line with our privacy policy, accessible here: https://goo.gl/7wrsDF


By selecting the "Start the survey" option below you agree to participate in the survey by The Mix to help us improve. You agree that you are 13 years old or older. ***

|  | Start the survey |
| --- | --- |
|  | I am 12 years old or younger |

**Total number of Signposts**

|  |
| --- |

**Signpost 1 Number**

|  |
| --- |

**Signpost 1 Name**

|  |
| --- |

**Signpost 2 Number**

|  |
| --- |

**Signpost 2 Name**

|  |
| --- |

**Signpost 3 Number**

|  |
| --- |

**Signpost 3 Name**

|  |
| --- |

**2. Thank you**

Thank you for your interest. We are sorry but due to GDPR regulations and your age we are unable to capture your data.

**Thinking about the last time you used The Mix helpline, please tell us how strongly you agree or disagree with the following statements. ***

|  | Strongly disagree | Tend to disagree | Tend to agree | Strongly agree | Don't know | Not applicable |
| --- | --- | --- | --- | --- | --- | --- |
| I felt listened to. |  |  |  |  |  |  |
| I felt understood. |  |  |  |  |  |  |
| I felt emotionally supported. |  |  |  |  |  |  |

**What could we have done to support you even better?**

|  |
| --- |

**How many times did you attempt to contact the helpline before you succeeded in getting through? ***

|  | Only once |
| --- | --- |
|  | 2 |
|  | 3 |
|  | 4 |
|  | 5 or more |
|  | Other (please specify):   \|  \| \| --- \| |

**Before you used the helpline were you clear about what to expect from the service? ***

|  | Yes |
| --- | --- |
|  | No |
|  | I don't know |

**Please tell us what was unclear**

|  |
| --- |

**How strongly you agree or disagree with the following statements: As a result of contacting the helpline... ***

|  | Strongly disagree | Tend to disagree | Tend to agree | Strongly agree | Don't know | Not applicable |
| --- | --- | --- | --- | --- | --- | --- |
| ...my well-being improved. |  |  |  |  |  |  |
| ...I feel more able to cope with my situation or issue. |  |  |  |  |  |  |
| ...I feel more capable to make decisions. |  |  |  |  |  |  |
| ...I formed/ I will form a plan to make a positive change to my situation. |  |  |  |  |  |  |

**Were any organisations, services or information sources recommended to you by the helpline agent? ***

|  | Yes |
| --- | --- |
|  | No |
|  | I don't know |

**Did you find the below organisations or resources recommended to you useful?**

|  | Yes | No | I haven't used it but I'M planning to do so | I haven't used it and NOT planning to do so | Not relevant |
| --- | --- | --- | --- | --- | --- |
| [variable(sp1name)] |  |  |  |  |  |

**Did you find the below organisations or resources recommended to you useful?**

|  | Yes | No | I haven't used it but I'M planning to do so | I haven't used it and NOT planning to do so | Not relevant |
| --- | --- | --- | --- | --- | --- |
| [variable(sp1name)] |  |  |  |  |  |
| [variable(sp2name)] |  |  |  |  |  |

**Did you find the below organisations or resources recommended to you useful?**

|  | Yes | No | I haven't used it but I'M planning to do so | I haven't used it and NOT planning to do so | Not relevant |
| --- | --- | --- | --- | --- | --- |
| [variable(sp1name)] |  |  |  |  |  |
| [variable(sp2name)] |  |  |  |  |  |
| [variable(sp3name)] |  |  |  |  |  |

**How would you rate the helpline service overall? ***

| Excellent | Good | Satisfactory | Poor | Very poor |
| --- | --- | --- | --- | --- |
|  |  |  |  |  |

**Did the service help you? ***

|  | Yes |
| --- | --- |
|  | No |

**Please tell us how the service helped you.**

|  |
| --- |

**How can we improve?**

|  |
| --- |

**Please let us know if you have any other comments or feedback.**

|  |
| --- |

**We’d like to use anonymous quotes from this survey on The Mix website or in reports to enable other people to better understand the service. Please tick the option below if we can use anonymous quotes from this survey.... (Please select all that apply). ***

|  | On The Mix website |
| --- | --- |
|  | In reports |
|  | None of the above |

**Can we contact you to send you the prize if you won? ***

|  | **Yes** |
| --- | --- |
|  | **No** |

# Section S2

Qualitative Codebook

**Theme 1: Experiences with Service Delivery**

Definition

Sentiments regarding the effectiveness of helpline delivery and execution by volunteers.

What Theme 1 is NOT

- Feedback regarding helpline protocol and standard practice (see Theme 2)
- Feedback on technical aspects or improvements of the service (see Theme 3)
- How users felt helped by the service (see Theme 4)

| **Code** | **Sub-Theme** | **Definition** | **Examples** |
| --- | --- | --- | --- |
| 1A | Delivery and Tone | Pace, tone, and language (including encouraging/empathetic phrases) used by volunteers during conversation with users | “make it less formal. Make it seem as if it’s a genuine conversation…”  “Giving us time to talk through our problems without rushing ” |
| 1B | Conversational Elements | Effectiveness of the type and number of questions asked by volunteers, conversational practices (e.g. rephrasing and repeating) | “I think it would of been better if the person asked a few more questions…”  “Perhaps not echo what I say back, I do understand why it’s done but can get a bit repetitive in the conversation” |
| 1C | Rushing and Rejection | Sentiments of feeling pushed away, misunderstood or excluded  Could be due to excessive signposting, insufficient understanding on matters, inability to cater to certain populations etc. | “Support people regardless of location”  “Provide a better understanding when reading someone’s issues. I felt like I wasn’t heard at all.”  “Try not to solely rely on signposting loads of organisations, as it can feel a bit demoralising to just be moved onto someone else…” |
| 1D | Responsiveness | How efficient users found the service, and how quickly, or whether, they received responses from volunteers (wait times) | “Faster response”  “You only got back to me once.” |

**Theme 2: Feedback on Service Protocol**

Definition

Sentiments regarding service protocol and standard practice by which the helpline is run.

What Theme 2 is NOT

- Feedback on how the service was delivered by volunteers (see Theme 1)

| **Code** | **Sub-Theme** | **Definition** | **Examples** |
| --- | --- | --- | --- |
| 2A | Lack of Personal Input and Concrete Support | Lack of personal opinions from volunteers, and practical advice regarding solutions or action plans | “I would like support with making a plan for tackling crises, giving myself the confidence to carry it out.”  “Own personal opinion if asked for advice.” |
| 2B | Alternative Support | Preference for longer-term follow-up support or face-to-face support | “Check in afew days after to see if things improved”  “See in person.” |
| 2C | Talk Times | Preference for longer talk times and less restriction on chat duration | “Probably how long you can talk to someone it Told me it ran out which can be frustrating…”  “Maybe just a bit more time for each agent, it felt too apparent when we hit the hour mark” |

**Theme 3: Feedback on Service Technicalities**

Definition

Sentiments regarding technical and administrative aspects of the service, including opening hours, IT matters and advertisement.

What Theme 3 is NOT

- Feedback regarding the delivery of the service by volunteers (see Theme 1)
- Feedback regarding practices/protocols adopted by the service (see Theme 2)

| **Code** | **Sub-Theme** | **Definition** | **Examples** |
| --- | --- | --- | --- |
| 3A | Opening Hours | Preference for keeping the helpline open for extended periods of time | “the 1-2-1 chat would be great if the opening times were extended “  “Longer opening times for the web chat!!” |
| 3B | Platform Functionality | Ensuring platforms are running without technical issues, and suggestions for improving platform functionality | “Not cut off”  “Have a traffic light system or estimated time to see how busy the helpline is. Have a button to save progress if you have to leave the chat unexpectedly.” |
| 3C | Advertising | Extending advertising of the service to wider audiences, and providing better/clearer information on how the service works | “I think its a great website and network the only improvemt would be to make it more well known to more people”  “Mention on the website what to expect when someone calls the helpline” |

**Theme 4: Type of Help Received**

Definition

Ways in which users felt helped (if they did) by the service.

What Theme 3 is NOT

- How effective users found the service delivery or protocol (see Theme 1 and Theme 2)

| **Code** | **Sub-Theme** | **Definition** | **Examples** |
| --- | --- | --- | --- |
| 4A | Felt Heard | Felt listened to, understood, and cared for without judgement | “I was listened to and was understood”  “they cared about me” |
| 4B | Someone to Talk to | Felt less lonely or benefitted from venting about and talking through their issues with someone | “Having someone to talk to”  “it helped me to know i’m not alone” |
| 4C | Better Understanding of Emotions and Circumstances | Made sense of their emotions, or gained new perspective regarding their situation | “Helped me let my thoughts out and make sense of them…”  “understand my problems better” |
| 4D | Empowerment and Direction | Felt more optimistic, confident and clear on how to seek help or move forward from their situation | “...I feel a lot more confident in myself and I had the courage to speak up to someone about how I felt, even if it was someone over the phone without face to face contact”  “It has helped me to try and see the positive in life” |
| 4E | Timely Support | Acute and timely support, practically (e.g. emergency services) or emotionally (e.g. calming down) | “Called the police”  “made me not want to cut myself”  “...I was at the darkest point and just desperately needed someone to hear me and that’s what he did. I got given other resources, as well as reccomendations and I came away from the chat feeling calmer and like I could get through another day.” |

| **Theme** | **No.** | **Sub-Theme** | **Definition** |
| --- | --- | --- | --- |
| 1. Experiences with Service Delivery | 1A | Delivery and Tone | Pace, tone, and phrases used by volunteers during conversation with users |
|  | 1B | Conversational Elements | Effectiveness of the type and number of questions asked by volunteers, conversational practices (e.g. rephrasing and repeating) |
|  | 1C | Rushing and Rejection | Sentiments of feeling pushed away, unheard, misunderstood or excluded  Could be due to excessive signposting, insufficient understanding on matters, inability to cater to certain populations |
|  | 1D | Responsiveness | How efficient users found the service, and how quickly, or whether, they received responses from volunteers (wait times) |
| 2. Feedback on Service Protocol | 2A | Lack of Personal Input and Concrete Support | Lack of personal opinions from volunteers, and practical advice regarding solutions or action plans |
|  | 2B | Alternative Support | Preference for longer-term follow-up support or face-to-face support |
|  | 2C | Talk Times | Preference for longer talk times and less restriction on chat duration |
| 3. Feedback on Service Technicalities | 3A | Opening Hours | Preference for keeping the helpline open for extended periods of time |
|  | 3B | Platform Functionality | Ensuring platforms are running without technical issues, and suggestions for improving platform functionality |
|  | 3C | Advertising | Extending advertising of the service to wider audiences, and providing better/clearer information on how the service works |
| 4. Type of Help Received | 4A | Felt Heard and Supported | Felt listened to, understood, and cared for without judgement |
|  | 4B | Someone to Talk to | Felt less lonely or benefitted from venting about and talking through their issues with someone |
|  | 4C | Better Understanding of Emotions and Circumstances | Made sense of their emotions, or gained new perspective regarding their situation |
|  | 4D | Empowerment and Direction | Felt more confident, optimistic and clear on how to seek help or move forward from their situation |
|  | 4E | Timely Support | Acute and timely support, practically (e.g. emergency services) or emotionally (e.g. calming down) |
